# UHPLC-MS-Based Metabolomics Analysis Reveals the Process of Schistosomiasis in Mice

**DOI:** 10.3389/fmicb.2020.01517

**Published:** 2020-07-14

**Authors:** Yuzheng Huang, Qiong Wu, Liang Zhao, Chunrong Xiong, Yongliang Xu, Xin Dong, Yan Wen, Jun Cao

**Affiliations:** ^1^National Health Commission Key Laboratory of Parasitic Disease Control and Prevention, Jiangsu Provincial Key Laboratory on Parasite and Vector Control Technology, Jiangsu Institute of Parasitic Diseases, Wuxi, China; ^2^Center for Global Health, School of Public Health, Nanjing Medical University, Nanjing, China; ^3^Public Health Research Center, Jiangnan University, Wuxi, China; ^4^Department of Pharmacy, General Hospital of Southern Theater Command, Guangzhou, China; ^5^Bloomberg-Kimmel Institute for Cancer Immunotherapy, The Sidney Kimmel Comprehensive Cancer Center at Johns Hopkins, Baltimore, MD, United States; ^6^School of Medicine, Shanghai University, Shanghai, China; ^7^Institute of Translation Medicine, Shanghai University, Shanghai, China; ^8^Department of Pharmacy, Changzheng Hospital, Second Military Medical University, Shanghai, China

**Keywords:** UHPLC-MS, metabolomics, schistosomiasis, early diagnosis, serum

## Abstract

Metabolomics, as an emerging technology, has been demonstrated to be a very powerful tool in the study of the host metabolic responses to infections by parasites. Schistosomiasis is a parasitic infection caused by *schistosoma* worm via the direct contact with the water containing cercaria, among which *Schistosoma japonicum* (*S. japonicum*) is endemic in Asia. In order to characterize the schistosome-induced changes in the host metabolism and further to develop the strategy for early diagnosis of schistosomiasis, we performed comprehensive LC-MS-based metabolomics analysis of serum from mice infected by *S. japonicum* for 5 weeks. With the developed diagnosis strategy based on our metabolomics data, we were able to successfully detect schistosomiasis at the first week post-infection, which was 3 weeks earlier than “gold standard” methods and 2 weeks earlier than the methods based on ^1^H NMR spectroscopy. Our metabolomics study revealed that *S. japonicum* infection induced the metabolic changes involved in a variety of metabolic pathways including amino acid metabolism, DNA and RNA biosynthesis, phospholipid metabolism, depression of energy metabolism, glucose uptake and metabolism, and disruption of gut microbiota metabolism. In addition, we identified seventeen specific metabolites whose down-regulated profiles were closely correlated with the time-course of schistosomiasis progression and can also be used as an indicator for the worm-burdens. Interestingly, the decrease of these seventeen metabolites was particularly remarkable at the first week post-infection. Thus, our findings on mechanisms of host-parasite interaction during the disease process pave the way for the development of an early diagnosis tool and provide more insightful understandings of the potential metabolic process associated with schistosomiasis in mice. Furthermore, the diagnosis strategy developed in this work is cost-effective and is superior to other currently used diagnosis methods.

## Introduction

Schistosomiasis, as one of the most severe infectious diseases and the most devastating tropical parasitic disease, is caused by *schistosoma* worm via the direct contact with the water containing cercaria (Ribeiro-dos-Santos et al., 2006; Wilby et al., 2013; Huang et al., 2016a, b). This disease is endemic in tropical and sub-tropical areas including 76 countries in Asia, Africa, and Latin America. In the worldwide, it was estimated that about 779 million individuals were at risk of infection, which led the loss of an estimated 4.5 million disability-adjusted life years (Rai et al., 2009; Huang et al., 2019, 2020). Acute infection was the most serious hazard for human beings, it can cause fever, gastrointestinal symptom, liver and spleen enlargement. And the advanced schistosomiasis occur when the treatment incomplete or delayed (Morel et al., 2014), which can further cause cardiopulmonary diseases (Valois et al., 2014), bladder cancer (Vale et al., 2017), and some other fatal cancers (Herman et al., 2017; Hamid, 2019).

Currently, the Kato-Katz technique by detecting eggs in feces under microscope and the immunological approaches by detecting soluble antigens secreted from the hatching-eggs via the antigen-antibody reaction were the two main methods for diagnosis, however, none of these two methods is suitable for the early diagnosis (Wu J. et al., 2010; Huang et al., 2011). In addition, considering the frequent false negative and positive results these methods produce, there is an urgent need to develop new diagnosis method. Previous studies already showed the schistosomiasis caused alterations at the transcription and protein levels (Rai et al., 2009; Sousa et al., 2013; Gobert et al., 2015; Weerakoon et al., 2015), however, these changes have not been explored in the early stage of the infection. Therefore, comprehensively study the dynamic response of hosts with schistosomiasis will help us the better understanding of the mechanisms underlying the disease progression and shed light on the development of more suitable tools for early diagnosis of schistosomiasis.

Metabolites in body fluid could manifest the status of health or disease to a certain extent, and also could be used as clue in diagnosis and treatment of diseases. Metabolomics, is concerned with the metabolite composition of biological systems and its dynamic responses to both endogenous and exogenous stimuli are particularly suitable for exploring the holistic metabolic responses to infections (Wang et al., 2004). For the past decade, metabolomics, as an emerging technology, has been demonstrated to be a very powerful tool in the study of the host metabolic responses to infections by parasites (Legido-Quigley, 2010; Pacchiarotta et al., 2012). Currently, for most of the metabolomics studies related to parasitic infection, capillary electrophoresis mass spectrometry (CE-MS), and nuclear magnetic resonance (NMR) were the most widely used technologies for the analysis of the dynamic metabolite changes in this field (Garcia-Perez et al., 2010; Legido-Quigley, 2010; Shin et al., 2011). Previous works have demonstrated that parasitic infection is related with disrupted pathway of energy metabolism, immune responses, glucose uptake and metabolism, and gut bacteria metabolism. However, these studies were mostly based on the well-established late-stage *schistosoma* infection model. Up to date, only one investigation based on the combination of NMR spectroscopy and multivariate data analysis has been carried out to study the time-course metabolomic changes over 5 weeks in the *Schistosoma japonicum* infected mice model (Wu J. et al., 2010). As one of the most popular technologies used in metabolomics field, NMR has several advantages over other technologies which include non-destructive sample preparation, suitable for structural elucidation, good reproducibility, and quantitative aspects. However, the analysis of NMR spectra of complex mixtures has traditionally been limited. Due to its relatively low sensitivity, NMR technology is not appropriate for comprehensive metabolite profiling of large number of low-abundance metabolites, and may lose some meaningful metabolites. Compared to NMR technology, MS has many important advantages such as high sensitivity, high-throughput, capability of the identification of the components present in complex biological samples, and the capacity of identification of unknown and unexpected compounds. All of the above mentioned advantages make MS a very powerful technology for detecting hundreds of compounds in metabolomics field. Moreover, MS, when combined with high-performance liquid chromatography, particularly with ultra-high-performance liquid chromatography (UHPLC-MS), can enable metabolomics analysis with much higher resolution, and better analytical flexibility (Bouhifd et al., 2013; Zhou et al., 2016). In this study, a LC-MS-based metabolomics method was established and was applied to study metabolic changes in the serum from the *S. japonicum* infected mice over the time course of 5 weeks. Thus, our study is aimed to explore the mechanisms of host-parasite interaction during the disease process and pave the way for the development of an early diagnosis tool for schistosomiasis.

## Materials and Methods

### Experimental Animals

All animal studies were performed in accordance with the National Institutes of Health (NIH) guide for the Care and Use of Laboratory Animals. The experimental procedures were approved by the Ethical Committee for the Experimental Use of Animals at Jiangsu institute of Parasitic Diseases (Wuxi, China). Fifty-three BALB/c female mice (120–130 *g*) at the age of 5 weeks were purchased from Yangzhou university (Yangzhou, China). Six mice per cage were provided by standard laboratory conditions (temperature of 20–25°C, relative humidity of 55–65%, and 12 h/12 h light/dark cycle) with free access to water and standard chow for 1 week before modeling. The mice were randomly divided into six groups, including control group and model groups. The model groups were infected with 40 cercariae of *S. japonicum* through the shaved abdominal skin for 30 min and no other treatment or manipulation of the animals was involved, and the control group was exposed to physiological saline at similarly shaved abdominal skin and raised at the same time. The blood samples were collected from tail vein at 0, 1, 2, 3, 4, and 5 weeks after infection with *S. japonicum*, respectively.

### Chemicals and Reagents

HPLC-grade methanol and acetonitrile (ACN) were purchased from Merk (Darmstadt, Germany). Formic acid was obtained from Fluka (Buchs, Switzerland). Ultrapure water was prepared with a Milli-Q water purification system (Millipore, Bedford, MA, United States).

### Sample Collection and Preparation

The blood samples were taken from tail venous and collected in a 1.5 mL tube at room temperature for 1 h, then centrifuged at 4,000 rpm for 15 min. The supernatant (serum) was aliquoted and stored at −80°C until LC-MS analysis. All the experiments were operated at the same time every week. 300 μL methanol was added to each 100 μL aliquot of serum. After vigorous shaking for 1 min, the mixtures were centrifuged at 13,000 rpm for 15 min at 4°C to precipitate the protein. Then, the serum was transferred to a sampling vial, and transferred to an autosampler vial and an aliquot of 4 μL was injected for LC-MS analysis. An in-house quality control (QC) was prepared by pooling and mixing the same volume of each sample. The QC sample was run six times prior to the start of the analytical run to “condition” the system and analyzed after every 8 samples to check for system stability.

### Global Metabolite Profiling

UHPLC analysis was performed on Agilent 1290 Infinity LC system (Agilent, Germany). An ACQUITY UPLC HSS T3 column (2.1 mm × 100 mm, 1.7 μm, Waters, Milford, MA, United States) was used to separate the serum samples at 45°C with a flow rate of 0.4 ml/min. The mobile phase consisted of A, 0.1% formic acid and B, ACN modified with 0.1% formic acid. The gradient program was as follows: 100% A at 0–2 min, 100%–85% A at 2–10 min, 85%–70% A at 10–14 min, 70%–5% A at 14–17 min, 5% A at 17–19 min, and 5%-100% A at 19–20 min, followed by a 5-min column re-equilibration.

An Agilent 6530 Accurate Mass Quadrupole Time-of-Flight (Q-TOF) mass spectrometer (Agilent, Santa Clara, CA, United States) was adapted to detect ion peaks, and the detection was operated at a negative ion mode. The cone gas was nitrogen with a flow rate of 11 L/h. The following detection parameters were used: fragment voltage, 120 V; capillary voltage, 3.5 kV; gas temperature, 350°C; and source temperature, 120°C. The full MS scan mode was monitored at the mass range of 50–1000 m/z. In the analyzing process, 10 mM purine (m/z 121.0508), and 2 mM hexakis phosphazinen (m/z 922.0097) were used as internal standards to guarantee mass accuracy and reproducibility. The centroid data were collected from the instrument. Subsequently, a MS/MS experiment was performed and the experiment parameters were set as follows: MS spectrum acquisition rate, 2 spectra/s, MS/MS spectrum acquisition rate, 0.5 spectra/s; and medium isolation window, 4 m/z; and collision energy, 20 V.

### Data Handling

Data processing used the method previously published by our group with minor modification. The raw data in instrument specific format (.d) were converted to common data format (.mzData) files using a conversion software program (file converter program available in Agilent MassHunter Qualitative sofware), in which the isotope interferences were eliminated. The program XCMS (version, 1.40.0) was used for non-linear alignment of the data in the time domain and automatic integration and extraction of the peak intensities. XCMS parameters were default settings (major default parameters: profmethod = bin; method = matched Filter; and step = 0.1) except for the following: full width at half maximum (FWHM) = 8, bandwidth (bw) = 10, and snthresh = 5, due to narrower peaks obtained by the use of the column packed with 1.7 μm particles. The variables presenting in at least 80% of either group were extracted, and the variables with a retention time less than 0.5 min (near to the dead time) were excluded due to a high degree of ion suppression that they suffered. The resulting three-dimensional matrix, including retention time and *m/z* pairs (variable indices), sample names (observations), and normalized ion intensities (variables), was exported to multivariate data analysis. The normalized data was introduced to SIMCA-P V12.0 (Umetrics, Sweden) for principal component analysis (PCA) and partial least squares discriminant analysis (PLS-DA) after mean-centering and pareto scaling, a technique that increased the importance of low abundance ions without significant amplification of noise. The quality of the models was evaluated with the relevant *R*^2^ and *Q*^2^. One-way ANOVA was performed to reveal the statistical differences in the significance of variation among control (0 week) and post-infection (1, 2, 3, 4, and 5 week) groups. And the Tukey *post hoc* test was applied for comparisons of multiple groups. The differences were considered significant when *p* < 0.05 and VIP > 1.

## Results

### Identification of Infected Animals

Stool samples were examined by the Kato–Katz thick smear method every week after infection, all the animal in the model group were recognized as infection successful once egg found in the stool (Utzinger et al., 2001). Furthermore, the eggs of feces were also collected and counted from all the infected mice after 6 weeks post-infection, which showed 4083.94 ± 474.94 eggs per gram of feces. All the experiments were carried out by the skilled technicians.

### System Stability Assessment

**FIGURE 1:**
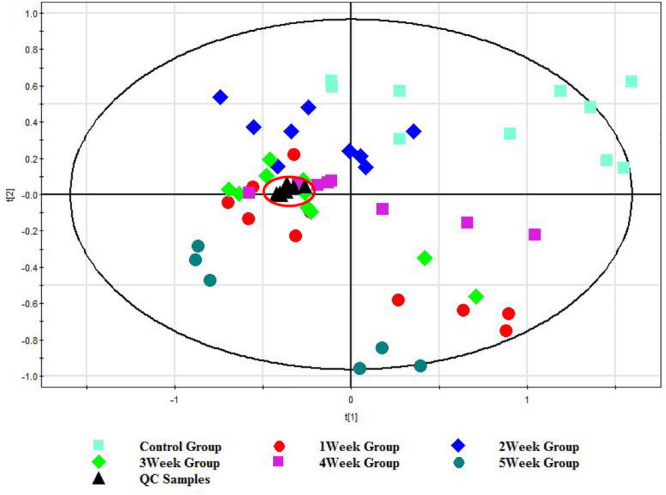
PCA score plot of the UHPLC/TOF-MS spectral from control group (0 w), post-infection group (1, 2, 3, 4, and 5 w), and QC group. Each color represents a group, and each point represents a sample. The farther the distance between the samples spots is, the more significant the difference between the samples is.

To validate the stability of the established method, an in-house QC sample was prepared by pooling and mixing the same volume of each sample. In order to present the real system state, QC data were introduced to SIMCA-P V12.0 for PCA after mean-centering and pareto scaling, as shown in Figure 1, all the QC samples were gathered together, which is a proof of system stability. The results indicated that the method was reliable for the subsequent analysis. In addition, the control and post-infection groups at different weeks were shown for some degree separation trend. The metabolic profiles of mice serum had an association with the time course of *S. japonicum* infection and disease progression. The metabolic profiles obtained from the infected mice deviated from the corresponding controls from the first week post-infection onwards and such separations became more obvious as disease progression. In order to identify the metabolites associated with such separations, we further compared the metabolic profiles obtained from the infected mice and corresponding controls for all matched time points, including the pre-infection day, week 1, 2, 3, 4, and 5 post-infection.

### Multivariate Statistical Analysis of Serum Metabolic Profiles

**FIGURE 2:**
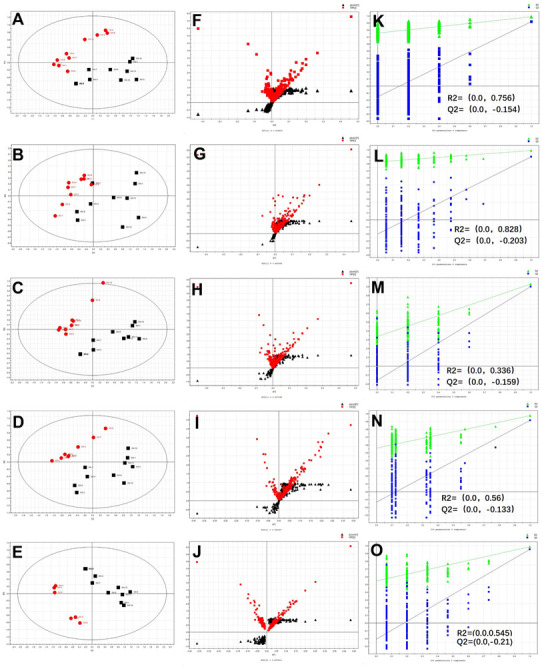
The results of multivariate data analysis. PLS-DA score map derived from UHPLC-Q-TOFMS spectra [**(A)** concerning 0 w and 1 w post-infection groups, **(B)** concerning 0 w and 2 w post-infection groups, **(C)** concerning 0 w and 3 w post-infection groups, **(D)** concerning 0 w and 4 w post-infection *n* groups, and **(E)** concerning 0 w and 5 w post-infection groups], red color represents post-infection groups (1, 2, 3, 4, and 5 w), block color represents control group; S-VIP plot [**(F)** concerning 0 w and 1 w post-infection groups, **(G)** concerning 0 w and 2 w post-infection groups, **(H)** concerning 0 w and 3 w post-infection groups, **(I)** concerning 0 w and 4 w post-infection groups, and **(J)** concerning 0 w and 5 w post-infection groups], red color represents variable importance in the projection, block color represents *p*(corr) vaule; Validation plot obtained from 200 permutation tests [**(K)** concerning 0 w and 1 w post-infection groups, **(L)** concerning 0 w and 2 w post-infection groups, **(M)** concerning 0 w and 3 w post-infection groups, **(N)** concerning 0 w and 4 w post-infection groups, **(O)** concerning 0 w and 5 w post-infection groups], green and blue colors represent *R*^2^ and *Q*^2^ values, respectively, used to evaluate whether the model is over-fitting.

The normalized data sets contained 1060 ions. To determine whether the metabolite fingerprints in serum deferred between the control and post-infection mice, we evaluated separation between the control and post-infection mice using supervised PLS-DA. The obvious separation was achieved between post-infection groups and control group, which were shown in Figures 2A–E. To validate the model, permutation tests with 200 iterations were further performed. These permutation tests compared the goodness of fit of the original model with the goodness of fit of randomly permuted models. As shown in Figures 2K–O, the validation plots indicates that the original models are valid. The criteria for validity are as follows: all the permuted *R*^2^ (cum) and Q*^2^* (cum) values to the left are lower than the original point to the right, and the blue regression line of the *Q*^2^ (cum) points has a negative intercept.

### Identification of Differential Serum Metabolites for *S. japonicum* Infection

Metabolites were carefully screened before being approved as potential biomarkers. First, significant original variables were extracted from the S-VIP plot, which is a covariance-correlation-based procedure, and thus the risk of false positives in metabolite selection was reduced. The S-VIP plot (Figures 2F–J), derived from the first component of the combined model, explains most of the variables in data set, in which the ions furthest away from the origin contribute significantly to the clustering of the two groups and may be regarded as potential biomarkers. Next, the variable importance for projection (VIP) indicating the importance of variables was applied to filter the important metabolites in the model. The most important 51, 54, 47, 58, 49 variables were first selected according to their VIP value when compared samples of 1, 2, 3, 4, and 5 week post-infection to those of 0 w, respectively.

One-way ANOVA were performed as the final testing procedure, and the critical *p*-value was set to 0.05 for significantly differential variables. Following the criterion above, 45, 44, 41, 49, and 48 metabolite ions were selected, respectively, as potential biomarkers related to *S. japonicum* infection at different time points. The detailed data was listed in Table 1.

**TABLE 1:** Summary of the potential biomarkers related to *S. japonicum* infection.

**No.**	**Compound**	**VIP^a^**	***P* value^a^**	**VIP^b^**	***P* value^b^**	**VIP^c^**	***P* value^c^**	**VIP^d^**	***P* value^d^**	**VIP^e^**	***P* value^e^**
1	9(S)-HpODE	1.04	< 0.001					1.24	< 0.001	1.00	< 0.001
2	D-Glucuronic acid	1.53	< 0.001	1.55	< 0.001	1.46	< 0.001	1.88	< 0.001	1.35	< 0.001
3	α-D-Glucose	5.74	0.029	6.03	0.016	6.24	0.002			6.10	0.005
4	19-Oxotestosterone	1.43	< 0.001					1.66	< 0.001	1.32	< 0.001
5	1-Methylinosine	1.17	< 0.001	1.44	< 0.001	1.20	< 0.001	1.75	< 0.001	1.22	< 0.001
6	3-Hydroxyphenyllactate	3.31	0.028	3.50	0.015	3.60	0.002			3.58	0.005
7	3-Sulfinoalanine	1.03	0.001	1.07	0.036			1.24	< 0.001		
8	5,6-Dihydroxyindole	1.72	0.014							1.85	< 0.001
9	Acetyl-DL-Valine	1.12	< 0.001			1.10	< 0.001				
10	Allopurinol	2.31	0.002	2.74	< 0.001	2.09	0.004	3.16	< 0.001	2.02	0.005
11	Anigorootin	1.21	0.029	1.30	0.01	1.03	0.015	1.48	0.002	1.10	0.024
12	Arachidonic acid	1.60	0.006								
13	Catechin 7-glucoside	1.54	0.04	1.69	0.008	1.74	0.005	2.11	0.016	1.74	0.009
14	Cerium	1.12	0.014	1.29	0.001	1.29	0.001	1.66	< 0.001	1.34	0.002
15	Cytidine monophosphate	1.09	< 0.001	1.05	< 0.001	1.05	< 0.001				
16	Deoxycholic acid 3-glucuronide	1.56	< 0.001	1.27	< 0.001	1.37	< 0.001	1.57	< 0.001	1.18	0.001
17	Dimethyl D-malate	1.59	< 0.001	1.68	< 0.001	1.24	0.004	1.67	< 0.001	1.04	0.042
18	Diphenol glucuronide	2.69	< 0.001	2.59	< 0.001	2.58	< 0.001	1.74	0.001	2.07	< 0.001
19	Glyceric acid	1.05	0.005	1.60	< 0.001	1.22	< 0.001	1.69	< 0.001	1.33	< 0.001
20	Glycerol tribenzoate	2.56	0.044	2.84	0.008	2.92	0.005	3.42	0.023	2.93	0.008
21	Hippuric acid	1.46	0.001					1.73	< 0.001	1.49	< 0.001
22	Hypoxanthine	2.67	0.001	1.97	0.048			2.90	0.001	2.15	0.005
23	Indole	1.96	0.033	1.27	0.005					1.85	0.008
24	Indoxylsulfuric acid	3.93	< 0.001			3.89	< 0.001	2.55	0.006	3.32	< 0.001
25	L-Phenylalanine	2.14	0.012	1.21	0.004	2.42	0.007			2.19	0.001
26	L-Tryptophan	3.23	< 0.001			3.16	< 0.001	2.63	0.001	2.64	< 0.001
27	L-Tyrosine	1.48	0.027	1.56	0.016	1.60	0.002			1.58	0.005
28	Muramic acid	2.23	0.03	2.66	0.002	2.53	0.002	3.61	< 0.001	2.23	0.012
29	N-Acetyl-D-glucosamine	1.91	< 0.001	1.82	< 0.001	1.81	< 0.001	2.01	< 0.001	1.49	< 0.001
30	N-Acetyl-L-tyrosine	1.08	< 0.001	1.44	< 0.001			1.09	< 0.001		
31	N-Acetylserotonin sulfate	1.16	0.005			1.16	0.001	1.26	< 0.001		
32	ND	1.87	0.012	2.15	< 0.001	2.13	0.001	2.69	< 0.001	2.18	0.002
33	ND	1.70	0.011	1.92	< 0.001	1.86	0.001	2.46	< 0.001	2.01	0.001
34	ND	1.50	0.012	1.69	0.001	1.65	0.001	2.17	< 0.001	1.77	0.002
35	ND	1.30	0.044	1.44	0.008	1.47	0.005	1.79	0.017	1.48	0.009
36	Oleic acid	2.14	0.005					2.13	0.035	1.97	0.002
37	PGE3	1.51	< 0.001	1.61	0.001	1.38	< 0.001	1.50	< 0.001	1.17	0.002
38	Prostaglandin A1	1.08	< 0.001	1.19	< 0.001	1.01	< 0.001	1.13	< 0.001		
39	PS(21:0/0:0)	2.90	< 0.001	2.37	< 0.001	2.56	< 0.001	2.92	< 0.001	2.17	0.001
40	Purine	1.18	< 0.001	1.34	< 0.001	1.14	< 0.001	1.15	< 0.001		
41	Pyrocatechol sulfate	1.48	< 0.001	1.25	0.013	1.38	< 0.001			1.25	0.001
42	Selenomethionine	1.75	0.001	2.18	< 0.001	1.66	< 0.001	2.28	< 0.001	1.77	< 0.001
43	Stearoyllactic acid	1.24	0.013	1.27	0.015			1.35	0.027	1.08	0.006
44	Thiamine	1.22	< 0.001			1.12	< 0.001	1.07	< 0.001		
45	Uridine	2.10	< 0.001	2.58	< 0.001	2.13	< 0.001	3.13	< 0.001	2.18	< 0.001
46	18-Hydroxyarachidonic acid			1.12	0.031			1.22	0.034	2.37	< 0.001
47	Dithionous acid			1.10	0.004			1.02	< 0.001		
48	D-Lactic acid			5.62	0.005	5.95	0.004			4.87	0.043
49	Formamide			1.27	0.004	1.12	0.002				
50	Isoplumbagin			1.13	< 0.001	1.08	< 0.001	1.05	< 0.001		
51	L-Threonine			2.03	0.011	1.74	0.01	1.22	0.004	2.02	< 0.001
52	Succinic acid			1.53	< 0.001	1.23	< 0.001	1.02	< 0.001	1.36	< 0.001
53	N-acetylaspartate							1.84	0.001	1.56	0.001
54	2-hydroxy-2-methyl-butyric acid			1.03	< 0.001						
55	Glucaric acid			1.08	0.008						
56	MG[18:2(9Z,12Z)/0:0/0:0]			1.03	0.002						
57	Deoxyuridine					1.24	0.003				
58	PGE3					1.38	< 0.001				
59	PE(18:0/14:0)					1.45	< 0.001				
60	(±)9-HODE							1.17	< 0.001		
61	(E)-13-Octadecenoic acid							1.01	0.041		
62	ADP-glucose							1.27	< 0.001		
63	Adrenosterone							1.06	< 0.001		
64	Avenoleic acid							1.16	< 0.001		
65	Deoxyinosine							1.17	< 0.001		
66	L-Arginine							1.27	< 0.001		
67	ND							1.13	< 0.001		
68	β-Alanine							1.08	< 0.001		
69	Cholic acid glucuronide									1.60	< 0.001
70	LysoPE[18:2(9Z,12Z)/0:0]									1.00	0.03
71	Myristoylglycine									1.07	0.001
72	Palmitoleic acid									1.04	0.008
73	Sinapinic acid-O-sulfate									1.06	< 0.001

*^*a*^Compared 1 w post-infection group with control group; ^*b*^Compared 2 w post-infection group with control group; ^*c*^Compared 3 w post-infection group with control group; ^*d*^Compared 4 w post-infection group with control group; and ^*e*^Compared 5 w post-infection group with control group. ND: not identified.*

### Pathway Enrichment Analysis of Serum Metabolic Profiles

**FIGURE 3:**
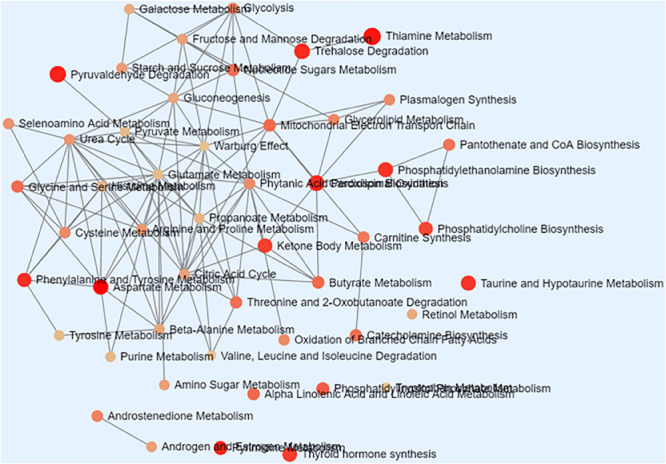
Schematic overview of the disturbed metabolic pathways associated with *Schistosoma japonicum* infection. Each node represents a metabolic pathway with its color based on its *p* value, and its size is based on fold enrichment to your query. Two pathways are connected by an edge if the number of their shared metabolites is over 25% of the total number of their combined metabolites.

As shown in Table 1, there were total 73 serum metabolites changed by *S. japonicum* infection over 5 weeks, which indicated that *S. japonicum* infection has disturbed the normal biological process in mice. In order to reveal the disturbed biological process, these identified serum metabolites were imported into **MetaboAnalyst 3.5** for functional enrichment analysis, and the detailed results were shown in Figure 3. 73 serum metabolites were mainly involved in aspartate metabolism, thiamine metabolism, glycerolipid metabolism, pyrimidine metabolism, pyruvaldehyde degradation, cardiolipin biosynthesis, trehalose degradation, phenylalanine and tyrosine metabolism, phosphatidylethanolamine biosynthesis, taurine and hypotaurine metabolism, ketone body metabolism, thyroid hormone synthesis, starch and sucrose metabolism, phosphatidylcholine biosynthesis, and phosphatidylinositol phosphate metabolism, etc.

**FIGURE 4:**
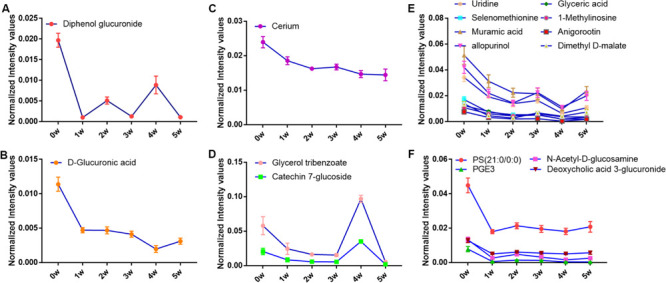
**(A)** The concentration changes of Diphenol glucuronide in *S. japonicum* infection mice. **(B)** The concentration changes of D-Glucuronic acid in *S. japonicum* infection mice. **(C)** The concentration changes of Cerium in *S. japonicum* infection mice. **(D)** The concentration changes of Glycerol tribenzoate and Catechin 7-glucoside in *S. japonicum* infection mice. **(E)** The concentration changes of Uridine, Selenomethionine, Muramic acid, allopurinol, Glyceric acid, 1-Methylinosine, Anigorootin, Dimethyl D-malate in *S. japonicum* infection mice. **(F)** The concentration changes of PS, PGE3, N-Acetyl-D-glucosamine, Deoxycholic acid 3-glucuronide in *S. japonicum* infection mice.

**FIGURE 5:**
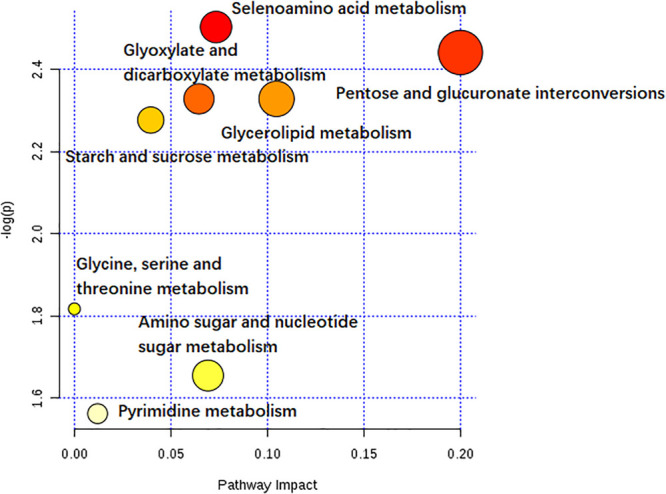
The pathway impact of *S. japonicum* infection based on the seventeen serum metabolites with MetaboAnalyst 3.5. Colors varying from yellow to red means the metabolites are in the data with different levels of significance.

Of all these 73 candidate metabolites in the above analysis, seventeen serum metabolites were finally screened and identified at all the five post-infection time points, including diphenol glucuronide, D-glucuronic acid, cerium, glycerol tribenzoate, catechin 7-glucoside, PS(21:0/0:0), N-Acetyl-D-glucosamine, PGE3, deoxycholic acid 3-glucuronide, uridine, selenomethionine, muramic acid, allopurinol, glyceric acid, 1-methylinosine, anigorootin, and dimethyl D-malate. The detailed data was listed in Table 2. And the levels of these metabolites were associated with the worm-burdens. In general, all the fluctuation trends can be classified into six categories, which is shown in Figure 4. The six trend types are important for uncovering the mechanism of *S. japonicum* infection and need to be deep mined and discussed in detail. Furthermore, pathway analysis with **MetaboAnalyst 3.5** revealed that the whole process of *S. japonicum* infection was involved in pentose and glucuronate interconversions, selenoamino acid metabololism, glycerolipid metabolism, glyoxylate and dicarboxylate metabolism, starch and sucrose metabolism, amino sugar and nucleotide sugar metabolism, pyrimidine metabolism, and glycine, serine and threonine metabolism, which were shown in Figure 5.

**TABLE 2:** Seventeen potential biomarkers identified at all five post-infection time points.

**No.**	**Mzmed**	**RT (min)**	**Compound**	**Related pathway**	**Trend ^1 w/0 *w*^**	**Trend ^2 w/1 w^**	**Trend ^3 w/2 w^**	**Trend ^4 w/3 w^**	**Trend ^5 w/4 w^**	**Type**
1	321.04	0.73	Diphenol glucuronide	ND	↓	↑	↓	↑	↓	A
2	193.04	4.96	D-Glucuronic acid	Pentose and glucuronate interconversions	↓	↓	↓	↓	↑	B
				Starch and sucrose metabolism						
3	174.83	1.80	Cerium	ND	↓	↓	↑	↓	↓	C
4	449.17	1.10	Glycerol tribenzoate	Glycerolipid metabolism	↓	↓	↓	↑	↓	D
5	451.17	1.10	Catechin 7-glucoside	ND	↓	↓	↓	↑	↓	
6	279.04	1.58	Uridine	Pyrimidine metabolism	↓	↓	↑	↓	↑	E
7	195.99	3.65	Selenomethionine	Selenoamino acid metabolism	↓	↓	↑	↓	↑	
8	286.06	5.38	Muramic acid	Amino sugar metabolism	↓	↓	↑	↓	↑	
9	135.03	3.95	Allopurinol	Bile secretion	↓	↓	↑	↓	↑	
10	105.02	3.70	Glyceric acid	Glycine, serine and threonine metabolism	↓	↓	↑	↓	↑	
				Glycerolipid metabolism						
				Glyoxylate and dicarboxylate metabolism						
11	281.04	1.60	1-Methylinosine	RNA biosynthesis	↓	↓	↑	↓	↑	
12	573.13	5.37	Anigorootin	ND	↓	↓	↑	↓	↑	
13	207.05	4.07	Dimethyl D-malate	Citric acid cycle	↓	↓	↑	↓	↑	
14	566.35	9.31	PS(21:0/0:0)	Phospholipid metabolism	↓	↑	↓	↓	↑	F
15	256.06	3.44	N-Acetyl-D-glucosamine	Amino sugar and nucleotide sugar metabolism	↓	↑	↓	↓	↑	
16	385.18	1.24	PGE3	Arachidonic acid metabolism	↓	↑	↓	↓	↑	
17	567.35	9.31	Deoxycholic acid 3-glucuronide	Bile acid metabolism	↓	↑	↓	↓	↑	

*A, B, C, D, E, and F: means six types of changing trends. ND: No metabolic pathways involved in this metabolite were found in this study.*

## Discussion

### Advantages of LC-MS-Based Metabolic Profiling of *S. japonicum* Infection at Early Stage

To the best of our knowledge, most of the previous metabolomic studies of *schistosoma* infections have been established on the end point of one schistosome life cycle when eggs have been produced. Only one investigation based on the ^1^H NMR spectroscopy in conjunction with multivariate data analysis has been reported which was focused on revealing the time-course metabonomic changes in the *S. japonicum* infection (Wu J. et al., 2010). This ^1^H NMR-based study has revealed metabolic changes from the third week post-infection in biofluids and liver samples, which means that the detection of infection can be achieved 1 week earlier than the current “gold standard” method. During this period, *S. japonicum* worms has not reached maturity or they won’t begin to lay eggs until around 4 weeks post-infection (Wilby et al., 2013). Furthermore, their studies indicated that the variations in metabolic profiles induced by the infection occurred before the sexual maturation of *S. japonicum* worms in the mammalian host and was thus prior to liver injuries by deposition of schistosomal eggs. In contrast to this study, our investigation based on LC-MS technology, as shown in Figure 2A, has revealed that the metabolic changes occurred from the first week post-infection in serum samples. This finding led to the detection of infection can be achieved 2 weeks and 3 weeks earlier than the previously reported ^1^H NMR-based metabolomic method and the current “gold standard” method, respectively. Therefore, the above mentioned findings from our study make it possible for the development of an early diagnostic tool for *S. japonicum* infection. Moreover, a total of 73 metabolites were identified and shown significant changes in metabolic profiles which were associated with the *S. japonicum* infection. The number of metabolites related to the infection we identified was almost two or three folds of those identified in the ^1^H NMR-based method. Our work has successfully demonstrated the usefulness of LC-MS-based metabolomics in parasitic infection studies. Furthermore, due to its high sensitivity, high-throughput, high resolution and good analytical flexibility, the LC-MS-based metabolomics method is well suitable for the comprehensive analysis of large number of metabolites and is superior to all other currently used analytical tools in this field.

### Disturbed Metabolic Pathways Associated With *S. japonicum* Infection

With the established LC-MS-based metabolomics method, we were able to identify a total of 73 metabolites with significant changes after *S. japonicum* infection. These metabolites were used to map on the metabolic pathways perturbed by the *S. japonicum* infection in mice model. To better understand the biological processes related to the infection, we performed pathway enrichment analysis based on these 73 serum metabolites. Figure 3 showed the enriched metabolic pathways associated with *S. japonicum* infection, which include the pathways involved in amino acid metabolism (aspartate metabolism, thyroid hormone synthesis), glucose uptake and metabolism (thiamine metabolism, trehalose degradation, taurine and hypotaurine metabolism, and starch and sucrose metabolism), phospholipid metabolism (glycerolipid metabolism, phosphatidylethanolamine biosynthesis, cardiolipin biosynthesis, phosphatidylcholine biosynthesis, and phosphatidylinositol phosphate metabolism), DNA and RNA biosynthesis (pyrimidine metabolism), energy metabolism (ketone body metabolism, pyruvaldehyde degradation), and gut microbiota ecology (phenylalanine and tyrosine metabolism). Liver injury is the typical symptom of *S. japonicum* infection in humans (Wu J-F. et al., 2010) and the disturbance of amino acid metabolism is one of the metabolic consequences of liver injury. Our findings regarding amino acid metabolism is consistent with the previous study. Another metabolic consequence of liver injury is the stimulated glycolysis, which was reflected by the remarkable reduction of glucose level in plasma following 5 weeks of infection (Wu J. et al., 2010). Interestingly, metabolite taurine, a cell membrane stabilizer and a potential conjugate with bile acid, was also found to be down-regulated after infection in our study, which was consistent with findings from other groups about the liver injury can cause decrease of taurine-conjugated bile acid production and further malabsorption in human with schistosomes infection (Wu J. et al., 2010; Wu J-F. et al., 2010). Phospholipids are playing important roles in maintaining the integrity and biological activities of liver cell membrane (Shinde et al., 2014; Uddin et al., 2014), the changes of phospholipid metabolism discovered in this study may imply the abnormalities of liver cell. All these metabolic findings showed the signs of liver dysfunction caused by *S. japonicum* infection in mice model. In order to promote their survial and transmission, Schistosomes have developed a very complex life cycle with discrete stages perfectly adapted to their differing hosts and free-living environments (Wang et al., 2016). Biological processes such as multiplication and proliferation in schistosomes are highly energy consuming, which are entirely reliant on the hosts for the essential nutrients required for development, reproduction and metabolism, For example, the blood flukes can take up glucose from their mammalian hosts and utilize its subsequent metabolism to fuel growth and fecundity (You et al., 2014). Again, the changes in glucose uptake and metabolism found in our study are correlated with infection progression and disease severity. Lipid metabolism is reported to be associated with the renewal of membrane complex, which is one of most abundant molecules present on the Schistosoma surface. Enough evidence has shown that lipids are essential in the life cycle of the parasite and they are playing an important role in the promotion of membrane fusion with the host resulting in the parasite acquisition of host membrane components. Moreover, these components are reported to have immune modulation properties and involved in maintaining lipid homeostasis in parasitic infection condition (Ferreira et al., 2014). Schistosomiasis is a disease caused predominantly by the host immune response to eggs and granulomatous reaction they evoke (Shinde et al., 2014; Chuah et al., 2016). The phospholipid metabolism may be closely related to the host’s immune response during parasitic infection. To our knowledge, thyroid hormones (TH) is essential for the regulation of growth, development and differentiation and they are actively involved in many metabolic processes by interacting with thyroid hormone receptors (THR) in the host (Qiu et al., 2012). Previous studies suggest that TH probably act indirectly or via pathways not involving the control of gene transcription for growth and development (de Mendonça et al., 2000). Therefore, the disturbed thyroid hormone synthesis found in our study is clearly consistent with these previous results, and these findings may provide important insights into the mechanism underlying host-parasite interactions during infection. Thiamine, also named Vitamin B1, is involved in the glucose metabolism and catabolism, and has a protective effect for nervous system. In this study, the thiamine metabolism was found to be disturbed after infection, which may suggest the possible abnormality in nervous system and the further confirmation of the disturbed glucose metabolism by schistosome infection in mice. Reports have been shown that gut microbiome can utilize phenylalanine and tyrosine to produce numerous metabolites that may regulate immune, metabolic, and neuronal responses at local and distant sites (Liu et al., 2020). The phenylalanine and tyrosine metabolism was also enriched in our study, which may indicate disturbance of the gut microbial ecology after schistosome infection, and this finding is consistent with the previous studies (Kay et al., 2015). Carboxylic acids, as the representatives of carbohydrate intermediary metabolism of both aerobic and anaerobic pathways, are directly linked to energy production and metabolism in both the host and the parasite (Abou Elseoud et al., 2010). While another central carbon metabolism, glycolysis, was reported to be the main energy resources for the parasite (Wu J. et al., 2010; Preidis and Hotez, 2015). Lactic acid, as a product of glycolysis, was found to be significantly higher in the serum of infected mice than in control mice, indicating that the glycolytic pathway of the infected mice was activated. This finding is also consistent with previous research conclusion that lactate was increased in schistosomiasis patients (Li et al., 2016). However, there is a difference in the conclusion that the host’s TCA cycle is inhibited after schistosomiasis infection (Tanabe et al., 1989; Ahmed and Gad, 1995). One of the TCA cycle intermediates, succinic acid, was found to be elevated in the serum of infected mice in this study, but the increase was not as strong as that of lactic acid. Except for this discrepancy, all other results indicated the active involvement of energy metabolism in the host reaction to the *S. japonicum* infection. In addition, pyrimidine metabolism was reported to play important roles in DNA and RNA biosynthesis (Tian et al., 2018; Zhao et al., 2018), therefore the changes in pyrimidine metabolism we identified in this study may suggest the potential effects on DNA and RNA biosynthesis caused by *S. japonicum* infection in mice.

### Potential Biomarkers for Early Diagnosis of *S. japonicum* Infection

As shown in Table 2, seventeen serum metabolites were finally screened and identified at all the five post-infection time points in this study. Although there are some fluctuations during the infection cycle, this may be associated with the worm-burdens and progression or the severity of the infections. In general, these metabolites were all decreased at five post-infection time points compared to the pre-infection groups. Notably, as shown in Figure 4, there was a significant decrease at the first week after *S. japonicum* infection. These changes could be indicative of the onset signals for the infection and these metabolites could be potential biomarkers for early diagnosis of *S. japonicum* infection. As reported in the previous study, uridine as a pyrimidine nucleoside, is the material basis of RNA synthesis, the bioavailability of which is particularly crucial to the synthesis of RNA and bio-membranes and the post-translational modification of protein (via the formation of pyrimidine nucleoside-lipid conjugates/UDP-sugar conjugates; Kurland et al., 2015; Cicuéndez et al., 2018; Tian et al., 2018). Uridine was also reported to exert protective effects against hepatotoxicity (Zhao et al., 2018). Therefore, the reduction of uridine in our study may indicate that the DNA and RNA synthesis was disturbed in the host and liver toxicity was induced by the infection (Zhou et al., 2018). It was reported that selenomethionine plays an important role in preventing oxidative stress and improving cell viability (Bai et al., 2019). Herein, the reduction of selenomethionine in our results may suggest that the stress status was induced in the host by infection. Phosphatidylserine (PS) was reported to play important roles in cellular apoptosis, and attract macrophages to engulf actions during tissue damage (Huang et al., 2016). Prostaglandin E3 (PGE3) as one of 3-series prostaglandin compounds, is the product of eicosapentaenoic acid (EPA) through cyclooxygenases (COXs) and possesses an anti-inflammatory effect (Yang et al., 2014; Wiktorowska-Owczarek et al., 2015; Gose et al., 2016). Cerium is also reported to exert anti-inflammatory and antioxidant effect (Serebrovska et al., 2017). As reported in the previous studies, parasite infection can lead to the formation of ROS and further contribute to oxidative stress in the host. Thus, the reduction of PGE3 and Cerium may be the consequences of its overuse to relieve inflammation. The reduction of catechin 7-glucoside, diphenol glucuronide, D-glucuronic acid and deoxycholic acid 3-glucuronide can reflect the disturbed phase II metabolism under infection condition, and the reduced phase II metabolism further confirmed the liver damage caused by infection. In addition, glyceric acid and glycerol tribenzoate are related with phospholipid synthesis, and phospholipid are essential membrane components for Schistosomes (Shinde et al., 2014). Dimethyl D-malate is the dimethyl conjugate of malate, which is the TCA cycle intermediates. Therefore, the reduction of phospholipid and TCA cycle intermediates may be the consequences of the overuse by parasites and induced by the activation of glycolysis. As a bacterial marker, muramic acid can serve as a core structural element for innate immune recognition of peptidoglycan (PG) fragments (Liang et al., 2017), the reduction of which may indicate the change in immune response in host after infection. Furthermore, pathway analysis with **MetaboAnalyst 3.5** revealed that these potential biomarkers are mainly involved in pentose and glucuronate interconversions, selenoamino acid metabololism, glycerolipid metabolism, glyoxylate and dicarboxylate metabolism, starch and sucrose metabolism, amino sugar and nucleotide sugar metabolism, pyrimidine metabolism, and glycine, serine and threonine metabolism (shown in Figure 5). In summary, these involved pathways provide more insightful understandings of the potential metabolic process associated with schistosomiasis. Furthermore, our findings on these mechanisms of host-parasite interaction during the disease process pave the way for the development of an early diagnosis tool.

## Data Availability Statement

All datasets generated for this study are included in the article/supplementary material.

## Ethics Statement

The animal study was reviewed and approved by the Animal Research Ethics Committee of the Jiangsu Institute of Parasitic Diseases.

## Author Contributions

YH, JC, and YW conceived and designed the experiments. YH, QW, LZ, CX, and YX performed the experiments. XD, YH, and QW analyzed the data. YH, YX, and JC collected the samples. All authors read and approved the final manuscript.

## Conflict of Interest

The authors declare that the research was conducted in the absence of any commercial or financial relationships that could be construed as a potential conflict of interest.

## References

[B1] Abou Elseoud S. M. Abdel Fattah N. S. Ezz El Din H. M. Abdel, Al H. Mossalem H. (2010). Carboxylic acids as biomarkers of Biomphalaria alexandrina snails infected with *Schistosoma mansoni*. *Korean J. Parasitol.* 48 127–132. 20585528 10.3347/kjp.2010.48.2.127 PMC2892567

[B2] Ahmed S. A. Gad M. Z. (1995). Effect of schistosomal infection and its treatment on some key enzymes of glucose metabolism in mice livers. *Arzneimittelforschung* 45 1324–1328. 8595093

[B3] Bai Z. Ren T. Han Y. Rahman M. M. Hu Y. Li Z. (2019). Influences of dietary selenomethionine exposure on tissue accumulation, blood biochemical profiles, gene expression and intestinal microbiota of Carassius auratus. *Comp. Biochem. Physiol. Part C* 218 21–29. 10.1016/j.cbpc.2018.12.001 30528703

[B4] Bouhifd M. Hartung T. Hogberg H. T. Kleensang A. Zhao L. (2013). Review: toxicometabolomics. *J. Appl. Toxicol.* 33 1365–1383. 10.1002/jat.2874 23722930 PMC3808515

[B5] Chuah C. Jones M. K. McManus D. P. Nawaratna S. K. Burke M. L. Owen H. C. (2016). Characterising granuloma regression and liver recovery in a murine model of schistosomiasis japonica. *Int. J. Parasitol.* 46 239–252. 10.1016/j.ijpara.2015.12.004 26812024

[B6] Cicuéndez M. Flores J. Oliveira H. Portolés M. T. Vallet-Regí M. Vila M. (2018). Metabolomic response of osteosarcoma cells to nanographene oxide-mediated hyperthermia. *Mat. Sci. Eng. C* 91 340–348. 10.1016/j.msec.2018.05.057 30033263

[B7] de Mendonça R. L. Escrivá H. Fau - Bouton D. Bouton D. Fau - Laudet V. Laudet V. (2000). Hormones and nuclear receptors in schistosome development. *Parasitol* 16 233–240. 10.1016/s0169-4758(00)01641-0 10827428

[B8] Ferreira M. S. de Oliveira D. N. de Oliveira R. N. Allegretti S. M. Vercesi A. E. Catharino R. R. (2014). Mass spectrometry imaging: a new vision in differentiating *Schistosoma mansonistrains*. *J. Mass Spectrom.* 49 86–92. 10.1002/jms.3308 24446267

[B9] Garcia-Perez I. Angulo S. Utzinger J. Holmes E. Legido-Quigley C. Barbas C. (2010). Chemometric and biological validation of a capillary electrophoresis metabolomic experiment of *Schistosoma mansoni* infection in mice. *Electrophoresis* 31 2338–2348. 10.1002/elps.200900523 20583011

[B10] Gobert G. N. Burke M. L. McManus D. P. Ellis M. K. Chuah C. Ramm G. A. (2015). Transcriptional profiling of chronic clinical hepatic *Schistosomiasis japonica* indicates reduced metabolism and immune responses. *Parasitology* 142 1453–1468. 10.1017/s0031182015000682 26216487

[B11] Gose T. Nakanishi T. Kamo S. Shimada H. Otake K. Tamai I. (2016). Prostaglandin transporter (OATP2A1/SLCO2A1) contributes to local disposition of eicosapentaenoic acid-derived PGE3. *Prostaglandins Other Lipid Mediat.* 122 10–17. 10.1016/j.prostaglandins.2015.12.003 26692285

[B12] Hamid H. K. S. (2019). *Schistosoma japonicum*-associated colorectal cancer: a review. *Am. J. Trop. Med. Hyg.* 100 501–505. 10.4269/ajtmh.18-0807 30560774 PMC6402928

[B13] Herman A. M. Kishe A. Babu H. Shilanaiman H. Tarmohamed M. Lodhia J. (2017). Colorectal cancer in a patient with intestinal schistosomiasis: a case report from Kilimanjaro Christian Medical Center Northern Zone Tanzania. *World J. Surg. Oncol.* 15:146. 10.1186/s12957-017-1217-1 PMC5541651 28768520

[B14] Huang H. Sun Z. Pan H. Chen M. Tong Y. Zhang J. (2016). Serum metabolomic signatures discriminate early liver inflammation and fibrosis stages in patients with chronic hepatitis B. *Sci. Rep.* 6:30853. 10.1038/srep30853 PMC4976343 27498553

[B15] Huang Y. Li W. Liu K. Xiong C. Cao P. Tao J. (2016a). Detection of sentinel mice using ClinProTool algorithm established by acute schistosomiasis japonica mice. *Parasitol. Res*. 115, 4173–4181. 27469535 10.1007/s00436-016-5193-0

[B16] Huang Y. Li W. Lu W. Xiong C. Yang Y. Yan H. (2016b) Cloning and characterization of a *Schistosoma japonicum* aquaglyceroporin that functions in osmoregulation. Sci. Rep. 6:35030. 10.1038/srep35030 PMC5062077 27733755

[B17] Huang Y. Lu J. Xu Y. Xiong C. Tong D Hu N. (2020). Xiaochaihu decorction relieves liver fibrosis caused by *Schistosoma japonicum* infection *via* the HSP47/TGF-β pathway. *Parasite Vectors* 13:254. 10.1186/s13071-020-04121-2 32410640 PMC7227055

[B18] Huang Y. Xu Y. Huang Y. Sun F. Tian H. Hu N. (2019). Identification of newly developed advanced schistosomiasis with MALDI-TOF mass spectrometry and ClinProTools analysis. *Parasite* 26:33. 10.1051/parasite/2019032 31166908 PMC6550559

[B19] Huang Y. Yang G. Kurian D. Xu M. Dai Y. Zhou Y. (2011). Proteomic patterns as biomarkers for the early detection of schistosomiasis japonica in a rabbit model. *Int. J. Mass Spectr*. 299, 191–195. 10.1016/j.ijms.2010.10.013

[B20] Kay G. L. Millard A. Sergeant M. J. Midzi N. Gwisai R. Mduluza T. (2015). Differences in the Faecal Microbiome in Schistosoma haematobium infected children vs. uninfected children. *PLoS Negl. Trop. Dis.* 9:e0003861. 10.1371/journal.pntd.0003861 26114287 PMC4482744

[B21] Kurland I. J. Broin P. Ó Golden A. Su G. Meng F. Liu L. (2015). Integrative metabolic signatures for hepatic radiation injury. *PLoS One* 10:e0124795. 10.1371/journal.pone.0124795 26046990 PMC4457483

[B22] Legido-Quigley C. (2010). Metabolite-biomarker investigations in the life cycle of and infection with Schistosoma. *Parasitology* 137 1425–1435. 10.1017/s0031182010000545 20550753

[B23] Li Y. Mei L. Qiang J. Ju S. Zhao S. (2016). Magnetic resonance spectroscopy for evaluating portal-systemic encephalopathy in patients with chronic hepatic *Schistosomiasis japonicum*. *PLoS. Negl. Trop. Dis.* 10:e0005232. 10.1371/journal.pntd.0005232 27977668 PMC5199111

[B24] Liang H. DeMeester K. E. Hou C.-W. Parent M. A. Caplan J. L. Grimes C. L. (2017). Metabolic labelling of the carbohydrate core in bacterial peptidoglycan and its applications. *Nat. Commun.* 8:15015. 10.1038/ncomms15015 PMC5411481 28425464

[B25] Liu Y. Hou Y. Wang G. Zheng X. Hao H. (2020). Gut microbial metabolites of aromatic amino acids as signals in host-microbe interplay. *Trends Endocrinol. Metab.* S1043-2760:30054-0. 10.1016/j.tem.2020.02.012 32284282

[B26] Morel M. Vanderstraete M. Cailliau K. Lescuyer A. Lancelot J. Dissous C. (2014). Compound library screening identified Akt/PKB kinase pathway inhibitors as potential key molecules for the development of new chemotherapeutics against schistosomiasis. *Int. J. Parasitol. Drugs Drug Resist.* 4 256–266. 10.1016/j.ijpddr.2014.09.004 25516836 PMC4266776

[B27] Pacchiarotta T. Deelder A. M. Mayboroda O. A. (2012). Metabolomic investigations of human infections. *Bioanalysis* 4 919–925. 10.4155/bio.12.61 22533566

[B28] Preidis G. A. Hotez P. J. (2015). The newest “Omics”-metagenomics and metabolomics-enter the battle against the neglected tropical diseases. *PLoS Negl. Trop. Dis.* 9: e0003382. 10.1371/journal.pntd.0003382 25675250 PMC4326130

[B29] Qiu C. Liu S. Hong Y. Fu Z. Wei M. Ai D. (2012). Molecular characterization of thyroid hormone receptor beta from Schistosoma japonicum and assessment of its potential as a vaccine candidate antigen against schistosomiasis in BALB/c mice. *Paras. Vect.* 5:172. 10.1186/1756-3305-5-172 22889153 PMC3438019

[B30] Rai G. Sayed A. A. Lea W. A. Luecke H. F. Chakrapani H. Prast-Nielsen S. (2009). Structure mechanism insights and the role of nitric oxide donation guide the development of oxadiazole-2-oxides as therapeutic agents against schistosomiasis. *J. Med. Chem.* 52 6474–6483. 10.1021/jm901021k 19761212 PMC2772170

[B31] Ribeiro-dos-Santos G. Verjovski-Almeida S. Leite L. C. (2006). Schistosomiasis–a century searching for chemotherapeutic drugs. *Parasitol. Res.* 99 505–521. 10.1007/s00436-006-0175-2 16636847

[B32] Serebrovska Z. Swanson R. J. Portnichenko V. Shysh A. Pavlovich S. Tumanovska L. (2017). Anti-inflammatory and antioxidant effect of cerium dioxide nanoparticles immobilized on the surface of silica nanoparticles in rat experimental pneumonia. *Biomed. Pharmacother.* 92 69–77. 10.1016/j.biopha.2017.05.064 28531802

[B33] Shin J. H. Yang J. Y. Jeon B. Y. Yoon Y. J. Cho S. N. Kang Y. H. (2011). (1)H NMR-based metabolomic profiling in mice infected with Mycobacterium tuberculosis. *J. Proteome Res.* 10 2238–2247. 10.1021/pr101054m 21452902

[B34] Shinde S. Mol M. Singh S. (2014). Regulatory networks, genes and glycerophospholipid biosynthesis pathway in schistosomiasis: a systems biology view for pharmacological intervention. *Gene* 550 214–222. 10.1016/j.gene.2014.08.031 25149020

[B35] Sousa T. N. D. Neto A. D. M. Brito C. F. A. D. (2013). “Omics” in the study of the major parasitic diseases malaria and schistosomiasis. *Infect. Genet. Evol.* 19 258–273. 10.1016/j.meegid.2013.07.008 23871773

[B36] Tanabe M. Kaneko N. Takeuchi T. (1989). Schistosoma mansoni: suppression of carbamoyl phosphate synthetase (ammonia) and ornithine carbamoyltransferase activities in the liver of infected mice. *Exp. Parasitol.* 68 432–442. 10.1016/0014-4894(89)90128-8 2498116

[B37] Tian Y. Wang Z. Liu X. Duan J. Feng G. Yin Y. (2018). Prediction of chemotherapeutic efficacy in non–small cell lung cancer by serum metabolomic profiling. *Clin. Cancer Res.* 24 2100–2109. 10.1158/1078-0432.ccr-17-2855 29437793

[B38] Uddin M. Fonseca C. S. M. D. Pimenta Filho A. A. Santos B. S. D. Silva C. A. D. Domingues A. L. C. (2014). Human plasma lipid modulation in *Schistosomiasis Mansoni* depends on apolipoprotein E polymorphism. *PLoS One* 9:e101964. 10.1371/journal.pone.0101964 25051269 PMC4106763

[B39] Utzinger J. Booth M. N’Goran E. K. Muller I. Tanner M. Lengeler C. (2001). Relative contribution of day-to-day and intra-specimen variation in faecal egg counts of Schistosoma mansoni before and after treatment with praziquantel. *Parasitology* 122 537–544. 10.1017/s0031182001007752 11393827

[B40] Vale N. Gouveia M. J. Rinaldi G. Santos J. Santos L. L. Brindley P. J. (2017). The role of estradiol metabolism in urogenital schistosomiasis-induced bladder cancer. *Tumor Biol.* 39:11. 10.1177/1010428317692247 28345469

[B41] Valois F. M. Nery L. E. Ramos R. P. Ferreira E. V. Silva C. C. Neder J. A. (2014). Contrasting cardiopulmonary responses to incremental exercise in patients with schistosomiasis-associated and idiopathic pulmonary arterial hypertension with similar resting hemodynamic impairment. *PLoS One* 9:e87699. 10.1371/journal.pone.0087699 24498356 PMC3912057

[B42] Wang T. Zhao M. Rotgans B. A. Strong A. Liang D. Ni G. (2016). Proteomic analysis of the *Schistosoma mansoni* miracidium. *PLoS One* 11:e0147247. 10.1371/journal.pone.0147247 26799066 PMC4723143

[B43] Wang Y. Holmes E. Nicholson J. K. Cloarec O. Chollet J. Tanner M. (2004). Metabonomic investigations in mice infected with *Schistosoma mansoni*: an approach for biomarker identification. *Proc. Natl. Acad. Sci.* 101 12676–12681. 10.1073/pnas.0404878101 15314235 PMC515115

[B44] Weerakoon K. G. A. D. Gobert G. N. Cai P. McManus D. P. (2015). Advances in the diagnosis of human *Schistosomiasis*. *Clin. Microbiol. Rev.* 28 939–967. 10.1128/cmr.00137-14 26224883 PMC4548261

[B45] Wiktorowska-Owczarek A. Berezińska M. Nowak J. Z. (2015). PUFAs: structures, metabolism and functions. *Adv. Clin. Exp. Med.* 24 931–941. 26771963 10.17219/acem/31243

[B46] Wilby K. J. Gilchrist S. E. Ensom M. H. H. (2013). A review of the pharmacokinetic implications of schistosomiasis. *Clin. Pharmacokinet.* 52 647–656. 10.1007/s40262-013-0055-8 23479397

[B47] Wu J. Xu W. Ming Z. Dong H. Tang H. Wang Y. (2010). Metabolic changes reveal the development of *Schistosomiasis* in mice. *PLoS Neg. Trop. Dis.* 4:e807. 10.1371/journal.pntd.0000807 20824219 PMC2930859

[B48] Wu J.-F. Holmes E. Xue J. Xiao S.-H. Singer B. H. Tang H.-R. (2010). Metabolic alterations in the hamster co-infected with Schistosoma japonicum and *Necator americanus*. *Int. J. Parasitol.* 40 695–703. 10.1016/j.ijpara.2009.11.003 19951707

[B49] Yang P. Jiang Y. Fischer S. M. (2014). Prostaglandin E3 metabolism and cancer. *Cancer Lett.* 348 1–11. 10.1016/j.canlet.2014.03.010 24657656 PMC4366418

[B50] You H. Stephenson R. J. Gobert G. N. McManus D. P. (2014). Revisiting glucose uptake and metabolism in schistosomes: new molecular insights for improved schistosomiasis therapies. *Front. Genet.* 5:176. 10.3389/fgene.2014.00176 24966871 PMC4052099

[B51] Zhao D.-S. Jiang L.-L. Wang L.-L. Wu Z.-T. Li Z.-Q. Shi W. (2018). Integrated metabolomics and proteomics approach to identify metabolic abnormalities in rats with *Dioscorea bulbifera* rhizome-induced hepatotoxicity. *Chem. Res. Toxicol.* 31 843–851. 10.1021/acs.chemrestox.8b00066 30052031

[B52] Zhou C.-X. Zhou D.-H. Elsheikha H. M. Zhao Y. Suo X. Zhu X.-Q. (2016). Metabolomic profling of mice serum during toxoplasmosis progression using liquid chromatography mass spectrometry. *Sci. Rep.* 20:13. 10.1038/srep19557 PMC4726199 26785939

[B53] Zhou K. Ding X. Yang J. Hu Y. Song Y. Chen M. (2018). Metabolomics reveals metabolic changes caused by low-dose 4-Tert-Octylphenol in mice liver. *Int. J. Environ. Res. Publ. Health* 15:2686. 10.3390/ijerph15122686 30487447 PMC6313621

